# PA from an H5N1 highly pathogenic avian influenza virus activates viral transcription and replication and induces apoptosis and interferon expression at an early stage of infection

**DOI:** 10.1186/1743-422X-9-106

**Published:** 2012-06-08

**Authors:** Qiang Wang, Shijian Zhang, Hongbing Jiang, Jinlan Wang, Leiyun Weng, Yingying Mao, Satoshi Sekiguchi, Fumihiko Yasui, Michinori Kohara, Philippe Buchy, Vincent Deubel, Ke Xu, Bing Sun, Tetsuya Toyoda

**Affiliations:** 1 Units of Molecular Virology, the Key Laboratory of Molecular Virology & Immunology, Institut Pasteur of Shanghai, Chinese Academy of Sciences, 411 Hefei Road, 200025, Shanghai, P. R. China; 2 Units of Viral Genome Regulation, the Key Laboratory of Molecular Virology & Immunology, Institut Pasteur of Shanghai, Chinese Academy of Sciences, 411 Hefei Road, 200025, Shanghai, P. R. China; 3 Shanghai Medical College of Fudan University, Yixueyuan Road 138, Shanghai, 200032, P. R. China; 4 Roche R&D Center China LTD, 720 Cai Lun Road, Building 5, Pudong, Shanghai, 201203, P. R. China; 5 Department of Microbiology and Cell Biology, Tokyo Metropolitan Institute of Medical Biology, 3-18-22 Honkomagome, Bunkyo-Ku, Tokyo, 113-8613, Japan; 6 Institut Pasteur in Cambodia, 5 Monivong Blvd, P.O. Box 983, Phnom Penh, Cambodia; 7 Choju Medical Institute, Fukushimura Hospital, 19-14 Azanakayama, Noyori-cho, Toyohashi, Aichi, 441-8124, Japan

**Keywords:** Influenza virus, PA, Transcription, Replication, Apoptosis, Interferon

## Abstract

**Background:**

Although gene exchange is not likely to occur freely, reassortment between the H5N1 highly pathogenic avian influenza virus (HPAIV) and currently circulating human viruses is a serious concern. The PA polymerase subunit of H5N1 HPAIV was recently reported to activate the influenza replicon activity.

**Methods:**

The replicon activities of PR8 and WSN strains (H1N1) of influenza containing PA from HPAIV A/Cambodia/P0322095/2005 (H5N1) and the activity of the chimeric RNA polymerase were analyzed. A reassortant WSN virus containing the H5N1 Cambodia PA (C-PA) was then reconstituted and its growth in cells and pathogenicity in mice examined. The interferon promoter, TUNEL, and caspase 3, 8, and 9 activities of C-PA-infected cells were compared with those of WSN-infected cells.

**Results:**

The activity of the chimeric RNA polymerase was slightly higher than that of WSN, and C-PA replicated better than WSN in cells. However, the multi-step growth of C-PA and its pathogenicity in mice were lower than those of WSN. The interferon promoter, TUNEL, and caspase 3, 8, and 9 activities were strongly induced in early infection in C-PA-infected cells but not in WSN-infected cells.

**Conclusions:**

Apoptosis and interferon were strongly induced early in C-PA infection, which protected the uninfected cells from expansion of viral infection. In this case, these classical host-virus interactions contributed to the attenuation of this strongly replicating virus.

## Background

Influenza A viruses cause disease in humans, pigs, other mammals, and birds [1]. The genomes of influenza A viruses are composed of 8 negative-sense single-stranded RNA segments; this segmented genome allows gene reassortment between viruses in co-infected cells to produce new viruses. Reassortment of influenza A virus genes caused the deadly H2N2 “Asian flu” and the H3N2 “Hong Kong flu” pandemics in 1957 and 1968, respectively. During these pandemics, the avian virus PB1, HA and NA, or PB1 and HA genes, respectively, were introduced into circulating human viruses [2,3]. The last pandemic strain, the novel swine-origin influenza virus A/H1N1 (S-OIV), carries PB2 and PA genes of avian origin [4]. In addition to the S-OIV pandemic flu, H5N1 highly pathogenic avian influenza viruses (HPAIV) have caused severe or fatal disease in humans in Asia, the Middle East, and Africa since their emergence in Hong Kong in 1997 (WHO, http://www.who.int/csr/disease/avian_influenza/en/). The H5N1 influenza virus ribonucleoprotein complex (RNP) contributes to viral pathogenesis in chickens [5,6]. Influenza viruses with high polymerase activity have also shown high pathogenicity [7,8]. These lines of evidence suggest that the influenza RNA-dependent RNA polymerase (RdRp) contributes to its pathogenesis.

Influenza A virions contain 8 negative-sense single-stranded RNA genome segments and RdRp [9]. Genome segments 1, 2, and 3 encode the RdRp subunits PB2, PB1, and PA, respectively. Segment 4 encodes hemagglutinin (HA), segment 5 nucleoprotein (NP), segment 6 neuraminidase (NA), segment 7 matrix protein 1 (M1) and an ion channel (M2), and segment 8 non-structural protein 1 (NS1) and nuclear export protein (NEP). A second open reading frame of segment 2 encodes PB1-F2 in some strains.

The RdRp subunit PB1 is the core subunit of the RdRp complex and mediates RNA polymerization [10-12], while PB2 is the cap-binding subunit [13-15] and PA has cap-dependent endonuclease activity [16-20]. Influenza virus RdRp catalyzes both transcription of viral mRNA and replication of the viral genome. Influenza transcription is a prototype of primer-dependent initiation. The viral RdRp binds to the cap-1 structures of host mRNAs and cleaves off 9 to 15 nucleotides to generate primers for viral transcription, a process known as “cap snatching” [13,21-23]. The internal initiation mechanism of influenza virus genome replication was recently elucidated [24,25].

H5N1 HPAIV does not efficiently adapt to transmission to humans [26]. However, once it does infect humans, it results in high mortality of approximately 60% [27,28]. Although some genomic combinations, such as those between human H3N2, avian H5N1, and horse H7N7 or between human H3N2 and avian H2N2, may be incompatible [29-32], reassortment between H5N1 HPAIV, which is epizootic among poultry almost worldwide [33], and currently circulating human influenza viruses, including the pandemic S-OIV and seasonal influenza viruses (H1N1 and H3N2), is one of the most important potential threats for the next pandemic. Kashiwagi *et al.* recently reported that the PA of H5N1 HPAIV activated the polymerase activity by enhancing promoter binding [34]. Multiple functions of PA in addition to promoter binding, such as transcription and replication [9,18], endonuclease activity [16,17,19,20,35], cap binding [19], protease activity [36], proteolysis induction [37], pathogenesis in mice [38], and thermal sensitivity of RNP [39], have been identified.

In this paper, we describe the activation of the polymerase activity of A/Puerto Rico/8/1934 (PR8, H1N1) and A/WSN/1933 (WSN, H1N1) RNPs by the H5N1 HPAIV PA of A/Cambodia/P0322095/2005, which was isolated from a Cambodian victim [40], and the reconstitution of the chimeric virus to analyze the effect of this H5N1 PA in the background of WSN, a well-studied mouse influenza infection model. We found a discrepancy between the viral polymerase activity and proliferation efficiency in cells and its pathogenesis in mice. We then analyzed the mechanism of the attenuation and the low pathogenicity of WSN carrying H5N1 PA.

## Results

### Effect of H5N1 Cambodia PA on the PR8 and WSN replicons and *in vitro* RdRp activity

**Figure 1:**
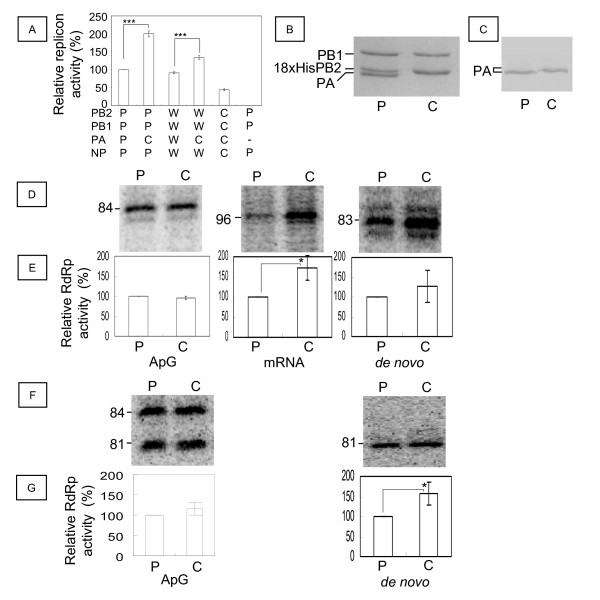
** Replicon and *in vitro *RNA-dependent RNA polymerase (RdRp) activities of the chimeras of H5N1 Cambodia PA with PR8 and WSN.A:** Replicon activity of the chimeric influenza virus ribonucleoproteins (RNPs) of WSN and PR8 with H5N1 Cambodia PA. The relative replicon activities of WSN and PR8 with H5N1 Cambodia PA in 293 T cells were compared with those of WSN and PR8. The combination of PB2, PB1, PA, and NP is indicated below the graph. W, WSN; P, PR8; C, H5N1 Cambodia. As a negative control, PR8 replicon without PA was used. **B:** Purified influenza virus PR8 RdRp (P) and PR8PB2-PR8PB1-C-PA RdRp (C). Each RdRp (5 pmol) was subjected to sodium dodecyl sulfate-polyacrylamide gel electrophoresis (SDS-PAGE) on 7.5% gels and Coomassie brilliant blue staining. The positions of PB1, 18 × His-PB2, and PA are indicated on the left. **C:** Western blot of the purified influenza virus chimeric RdRp. Each RdRp (5 pmol) was electro-blotted onto the nitrocellulose membrane. The PA was detected by western blotting with anti-PA antibodies. The position of PA is indicated on the left. **D** and **E:** Comparison of the chimeric RdRp activities in *in vitro* transcription and replication of v84 with those of PR8. The v84 model template RNA (200 nM) was transcribed by PR8RdRp (P) or PR8PB2-PR8PB1-C-PA RdRp (C) with or without (*de novo*) 0.1 mM ApG (ApG) or 0.5 μg globin mRNA (mRNA). **F** and **G:** Comparison of chimeric RdRp activity in *in vitro* replication of c84 with that of PR8. The c84 model template RNA was incubated with or without ApG (*de novo*). Products were analyzed by PAGE on 6% gels containing 8 M urea and the images analyzed with a Typhoon Trio plus. Examples of the images are shown in D and F. The size of the product RNA is indicated on the left. The mean and standard deviation (error bar in the graph) of the polymerase activity relative to that of PR8 RdRp were calculated from 2 independent measurements of 3 different RdRp preparations. Statistical significance was evaluated with Student’s *t*-test. *p < 0.05, ***p < 0.005.

We first examined the replicon activity in 293 T cells of a chimeric PR8 RNP containing H5N1 Cambodia PA (Figure 1A). Influenza replicon activity was measured as previously described [41,42]. The replicon activity was about 200.0 ± 8.2% that of the PR8 RNP (Student’s *t*; p < 0.005), while the Cambodia RNP showed 43.8 ± 2.9% of the replicon activity of the PR8 RNP (p < 0.005). The expression of RdRp and NP in the transfected cells was confirmed by western blotting (Additional file 1: Figure S1).

As we found 2-fold activation of the PR8 replicon by H5N1 Cambodia PA, we purified both PR8 RdRp and a chimeric RdRp (PR8PB2-PR8PB1-H5N1 Cambodia PA) in order to investigate the effect of the H5N1 Cambodia PA on RdRp activity *in vitro*. Sodium dodecyl sulfate-polyacrylamide gel electrophoresis (SDS-PAGE) showed that H5N1 Cambodia PA migrated to a higher position than did PR8PA; the Cambodia PA band almost overlapped with that of 18 × HisPR8PB2 (Figure 1B). The identity of H5N1 Cambodia PA was confirmed by western blotting against PA (Figure 1C).

We next compared the activities of the purified RdRps *in vitro*. We tested the transcription activity using v84 and globin mRNA primers (Figure 1D and E, mRNA), and replication activity using v84 and c84 with and without the dinucleotide primer ApG (Figure 1D, E, F, and G). ApG-primed and *de novo* initiation of v84 transcription produced 84- and 83-nt products, respectively, while globin mRNA-primed transcription produced 96-nt products. Because virion RdRp uses 10–15 nucleotide primers to initiate from the C at the 2nd position from the 3′ end of the genome [18,22], the 96-mer products were assigned to the transcripts from the 13th G next to the 12th U of the cap-1 structure (m^7^GmACACUUGCUUUU) of rabbit β-globin mRNA (GenBank; M10843). The mean and standard deviation (error bar in the graph) of the polymerase activity relative to that of PR8 RdRp were calculated from 2 independent measurements of 3 different RdRp preparations. The relative ApG-primed replication activity of the chimeric RdRp was 95.9 ± 4.5% of that of PR8 RdRp, while its *de novo* replication activity was 126 ± 40% of that of PR8 RdRp. The chimeric RdRp produced 171 ± 31% (p < 0.05) of the amount of 96-nt transcription products. ApG-primed initiation of c84 produced 84- and 81-nt products, while *de novo* initiation produced 81-nt products. The relative replication activity of ApG-primed initiation of c84 by the chimeric RdRp was 116 ± 16% of that by PR8 RdRp, while its *de novo* initiation was 156 ± 29% of that by PR8 RdRp (p < 0.05). We thus confirmed that H5N1 Cambodia PA enhanced both the transcription and replication activities of PR8 RdRp.

Before reconstituting WSN carrying H5N1 Cambodia PA, we tested the effect of H5N1 Cambodia PA on the WSN replicon (Figure 1A). WSN replicon activity (91.3 ± 3.2%) was similar to that of PR8. The replicon activity of WSN containing the H5N1 Cambodia PA (which was 133.9 ± 5.8% of that of the PR8 replicon) was about 1.5-fold higher than that of the WSN replicon (p < 0.005). Therefore, we also observed an activation effect of H5N1 Cambodia PA on the WSN replicon.

### Effect of H5N1 Cambodia PA on virus growth in MDCK cells, chicken embryo fibroblasts (CEF), and Vero cells

Next, we tested whether the RdRp activation effect of H5N1 Cambodia PA affected virus growth. As the RdRp subunits of PR8 and WSN are highly homologous, with 96, 97, and 98% amino acid identity between the PB2, PB1, and PA genes, respectively, and as the activation effect of H5N1 Cambodia PA was confirmed in both the WSN and PR8 replicons, we used a WSN reconstitution system to analyze the effect of H5N1 Cambodia PA in H1N1 virus. The WSN virus carrying H5N1 Cambodia PA (C-PA) was reconstituted successfully. First, we compared the multi-step growth of C-PA with that of WSN in MDCK, CEF, and Vero cells.

**Figure 2:**
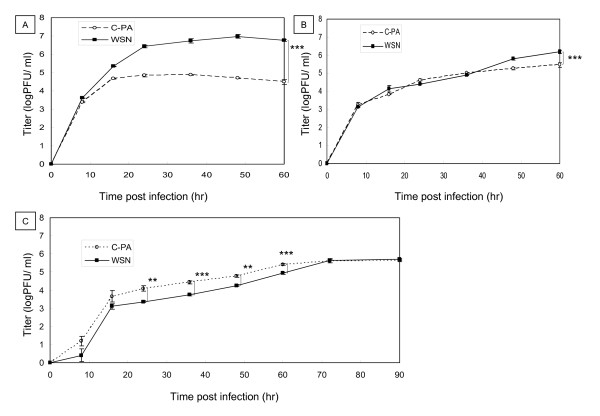
** Multi-step growth curves of C-PA and WSN in infected cells.** MDCK **(A)**, chicken embryo fibroblast (CEF) **(B)**, and Vero **(C)** cells were infected with C-PA or WSN at a multiplicity of infection (MOI) of 0.01. Viruses were harvested 8, 16, 24, 36, 48, and 60 hr (additional 72 and 90 hr for Vero cells) after infection and their titers on MDCK cells measured by a plaque-formation assay. The mean and standard deviation (error bar in the graph) of each plaque titer were calculated from 3 independent experiments. Statistical significance was evaluated with Student’s *t*-test. **p < 0.01, ***p < 0.005.

In MDCK cells, the C-PA titer was always lower than that of WSN and plateaued (4.7 ± 0.2 × 10^4^ PFU/mL) 16 hr post-infection (pi), while the WSN titer was 2.2 ± 0.03 × 10^5^ PFU/mL 16 hr pi and plateaued (5.6 ± 1.6 × 10^6^ PFU/mL) 36 hr pi (Figure 2A). The C-PA titer in MDCK cells 60 hr pi was significantly higher than that of WSN (p < 0.001). The titers of C-PA and WSN in CEF did not plateau even 60 hr pi. However, the C-PA titer 60 hr pi (7.1 ± 1.4 × 10^5^ PFU/mL) was about half of that of WSN (1.3 ± 0.4 × 10^6^ PFU/mL), a statistically significant difference (p < 0.001, Figure 2B). We therefore observed a discrepancy between the effects of H5N1 Cambodia PA on RdRp activity and on virus growth in MDCK and CEF cells. However, the C-PA titer in Vero cells between 24 hr and 60 hr pi was significantly higher than that of WSN (p < 0.05, Figure 2C). Both titers plateaued at 5.6 ± 0.1 × 10^5^ PFU/mL 70 hr pi. No cytopathic effect was apparent in Vero cells (data not shown).

**Figure 3:**
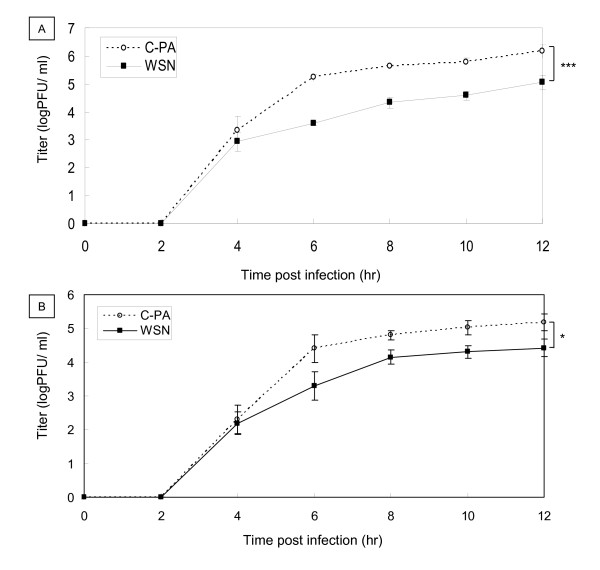
** Single-step growth curves of C-PA and WSN in infected cells.** MDCK **(A)** and Vero **(B)** cells were infected with C-PA or WSN at an MOI of 5. Viruses were harvested 2, 4, 6, 8, 10, and 12 hr after infection and their titers on MDCK cells measured by a plaque-formation assay. The mean and standard deviation (error bar in the graph) of each plaque titer were calculated from 3 independent experiments. Statistical significance was evaluated with Student’s *t*-test. *p < 0.05, ***p < 0.005.

We next compared single-step growth of C-PA and WSN in MDCK and Vero cells (Figure 3). The C-PA titers in both MDCK and Vero cells between 4 and 12 hr pi were higher than those of WSN. The C-PA titer in MDCK cells 12 hr pi (1.6 ± 0.9 × 10^6^ PFU/mL) was significantly higher than that of WSN (1.3 ± 0.7 × 10^5^ PFU/mL) (p < 0.005, Figure 3A). The C-PA titer in Vero cells 12 hr pi (1.6 ± 0.9 × 10^5^ PFU/mL) was significantly higher than that of WSN (2.9 ± 0.2 × 10^4^ PFU/mL) (p < 0.05, Figure 3B). The growth of C-PA in both MDCK and Vero cells plateaued 6 hr pi. WSN growth in Vero cells plateaued 8 hr pi, while its titer in MDCK cells continued to increase up to 12 hr pi.

### Pathogenicity of the C-PA virus in mice

**Figure 4:**
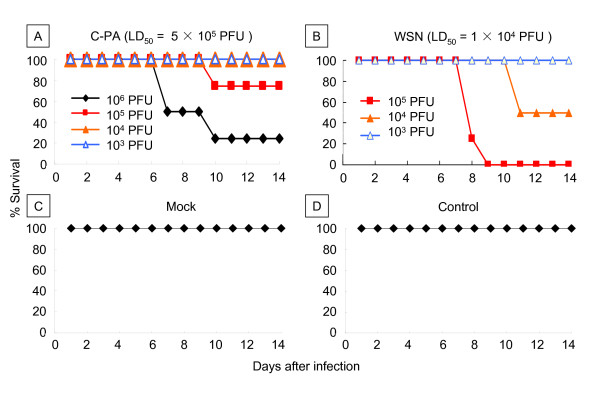
** Survival curve of mice infected with C-PA or WSN.** Groups of 4 mice were inoculated intra-nasally with 10^6^, 10^5^, 10^4^, or 10^3^ plaque-forming units (PFU) of C-PA **(A)** or 10^5^, 10^4^, or 10^3^ PFU of WSN **(B)** in a volume of 50 μL. The survival curves of the mock-infected **(C)** and non-treated (control) **(D)** mice are also shown. Survival status was monitored daily. LD_50_ is indicated above the graph (A and B).

We also examined the pathogenicity of C-PA in mice. The LD_50_ for mouse nasal infection was calculated from the survival rate of the infected mice by the method of Reed and Münch (Figure 4) [43]. The LD_50_ values of C-PA and WSN were 5 × 10^5^ PFU and 1 × 10^4^ PFU, respectively. The pathogenicity of C-PA is therefore lower than that of WSN, despite its higher RdRp activity, both in cell culture (as reflected by the multi-step growth) and in mice.

We thus confirmed the discrepancy between the RdRp activity and pathogenicity both in cells (virus titer) and in mice. The genome sequence of the C-PA stock was confirmed to be identical to that of the genome reconstitution plasmids.

### Interferon induction

**Figure 5:**
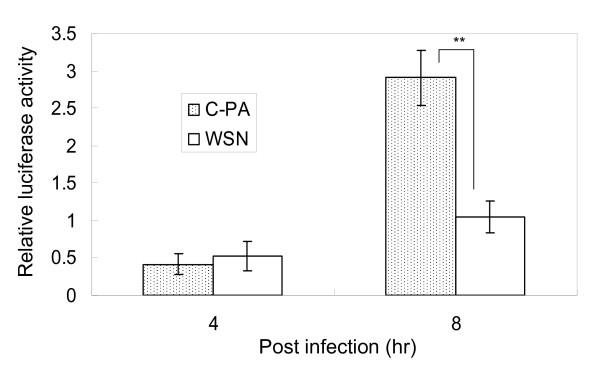
** Interferon β expression in C-PA- and WSN-infected cells.** Interferon induction levels 4 and 8 hr pi were measured as the luciferase activity from p-125Luc. The mean and standard deviation (error bar) of luciferase activity were calculated from 3 independent measurements. Statistical significance was evaluated with Student’s *t*-test. **p < 0.01.

Because the multi-step growth activity of C-PA was lower than that of WSN in MDCK and CEF cells but better in Vero cells, and because the single-step growth activity of C-PA was better than that of WSN in both MDCK and Vero cells, we examined the effect of C-PA on the activity of the host cellular defense system. First, we examined interferon β induction in 293 T cells by measuring the activity of its promoter (Figure 5). A small amount of interferon β was induced in cells infected with either C-PA or WSN 4 hr pi. However, interferon β promoter activity was almost 3-fold higher (p < 0.01) in the C-PA-infected cells than in the WSN-infected cells 8 hr pi.

### TUNEL assay

**Figure 6:**
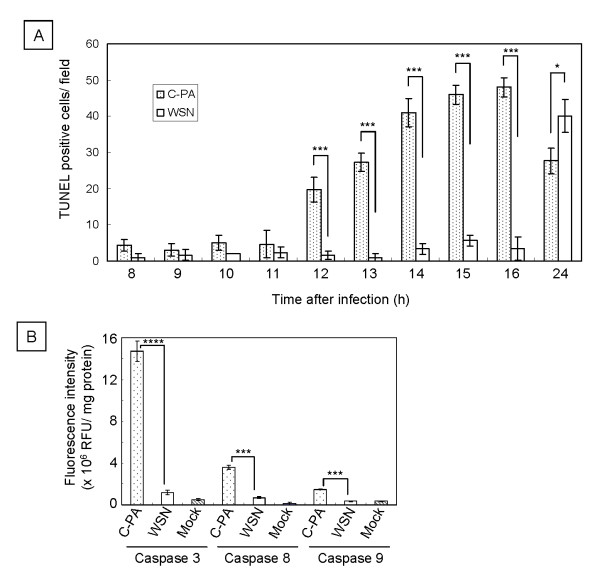
** TUNEL staining and Caspase 3, 8, and 9 activities of C-PA- and WSN-infected cells.A.** TUNEL assays were performed on C-PA- and WSN-infected MDCK cells at the indicated time after infection. The mean and standard deviation (error bar) of the number of TUNEL-positive cells counted from 3 random fields were calculated. **B.** The activities of caspases 3, 8, and 9 in C-PA- and WSN-infected MDCK cells were measured 10 hr pi. Caspase activity is expressed as the fluorescence intensity/total cellular protein (mg) calculated from 3 independent measurements. Mock: mock-infected MDCK cells. Statistical significance was evaluated with Student’s *t*-test. *p < 0.05, ***p < 0.005, ****p < 0.0005.

Next, we examined the induction of apoptosis in MDCK cells by performing TUNEL assays hourly from 8 to 16 hr pi and once more 24 hr pi. The numbers of TUNEL-positive cells in 3 random fields were counted (Figure 6A). Before 11 hr pi, we found fewer than 5 TUNEL-positive cells per field in either C-PA- or WSN-infected cells. Then, beginning 12 hr pi, the numbers of the C-PA-infected TUNEL-positive cells increased to 20 ± 3.5 (12 hr), 27 ± 2.5 (13 hr), 41 ± 4.0 (14 hr), 46 ± 2.6 (15 hr), 48 ± 2.6 (16 hr), and 28 ± 3.5 (24 hr). The numbers of the WSN-infected TUNEL-positive cells remained less than 6 until 16 hr and then increased to 40 ± 4.6 at 24 hr pi. From 12 to 16 hr pi, significantly more TUNEL-positive cells were observed among the C-PA-infected cells than among the WSN-infected cells (p < 0.005). TUNEL-positive cells were clearly observed among both C-PA- and WSN-infected cells, and significantly more TUNEL-positive cells were observed among WSN-infected cells than among C-PA-infected cells 24 hr pi (p < 0.05). In conclusion, TUNEL-positive cells appeared earlier in C-PA-infected cells than in WSN-infected cells. No differences in TUNEL staining were found among cells transfected with WSN and H5N1 Cambodia single-protein-expression plasmids (PB2, PB1, PA, HA, NP, NA, M1, M2, NS1, and NS2) or pcDNA3.1 (data not shown).

### Caspase 3, 8, and 9 activities

TUNEL-positive cells may be apoptotic. Caspases are central players in both the intrinsic and the extrinsic pathways of apoptosis [44-46]. We therefore measured the activities of caspases 3, 8, and 9 in C-PA- and WSN-infected cells 10 hr pi, 2 hr before TUNEL-positive cells could first be observed among C-PA-infected cells, in order to confirm the induction of apoptosis (Figure 6B). The activities of caspases 3 (14.7 ± 9.8 × 10^6^ RFU/mg protein, p < 0.0005), 8 (3.6 ± 0.2 × 10^6^ RFU/mg protein, p < 0.005), and 9 (1.5 ± 0.5 × 10^6^ RFU/mg protein, p < 0.005) were higher in C-PA-infected cells than in WSN-infected cells 10 hr pi (comparisons evaluated by Student’s *t* test). The caspase activities in WSN-infected cells were similar to those in mock-infected cells, indicating that WSN infection did not induce apoptosis 10 hr pi. The activation of caspase 9 in C-PA-infected cells indicates that C-PA infection induces apoptosis through the mitochondrial pathway [47-49], and the apoptosis induction by C-PA began at an early time after infection at which no apoptosis was induced by WSN infection.

### Histopathology and TUNEL assay of infected mice

**Figure 7:**
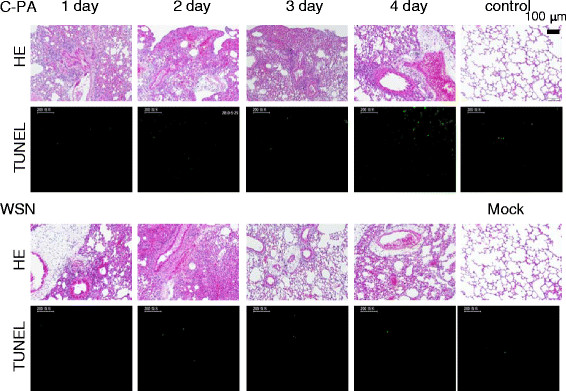
** Histopathology of C-PA- and WSN-infected mouse lungs.** The lungs of mice infected with C-PA or WSN (10^5^ PFU/mouse) at the indicated days after infection were imaged after hematoxylin and eosin (HE) or TUNEL staining. Control: non-infected mouse lung. The bar above the picture indicates 100 μm and the bars in the fluorescence microgram indicate 200 μm.

Finally, we compared the pathological changes in the lung between C-PA- and WSN-infected mice. The histopathological appearances were similar (Figure 7). The major difference between C-PA- and WSN-infected lungs is that pulmonary edema around blood vessels was present in the C-PA-infected lungs from day 1 pi, although it was also present in WSN-infected lungs on days 3 and 4 pi. On the first day of infection, a moderate amount of lymphocyte infiltration was observed around the bronchioles of C-PA-infected mouse lungs, and this inflammation decreased on days 3 and 4. More lymphocyte infiltration around the bronchioles was observed in WSN-infected mouse lungs on days 1–3, with the most severe inflammation on day 2, and only mild inflammation was observed on day 4. No pathological change was observed in the mock-infected or non-infected mouse lungs.

We simultaneously analyzed these samples by TUNEL assay. A few TUNEL-positive cells were observed in both C-PA- and WSN-infected mouse lungs on days 1–4 pi, but the numbers did not clearly differ between the 2 viruses. The histopathological findings indicated recovery of the C-PA-infected mouse lungs, which is consistent with the lower pathogenicity of C-PA.

## Discussion

The activation of replicon activity by H5N1 HPAIV PA observed in this study has been reported several times (Figure 1) [34,50,51]. Influenza viruses with high polymerase activity have been reported to show high pathogenicity [7,8]. Therefore, reassortment of H5N1 HPAIV PA into human influenza viruses, including the seasonal influenza viruses (H1N1 and H3N2) and the pandemic influenza virus (H1N1), is a serious concern [34], although this event may not occur easily [32]. The major factor for host adaptation in H5N1 HPAIV RNP is PB2, which shows a strong correlation with pathogenicity in mammalian hosts, including humans [30,31,51-55].

The WSN reconstitution system [56] was used to analyze the effect of H5N1 Cambodia PA in an animal influenza virus (H1N1). Contrary to our expectation, the multi-step growth of C-PA in MDCK and CEF cells was less than that of WSN, and its pathogenicity was low because of this attenuation (Figures 2,4). We confirmed the absence of mutations in the C-PA genome. As we previously found a similar discrepancy in influenza promoter/origin function due to a difference in activation of the host defense system [42], we examined whether the discrepancy between RdRp activity and pathogenicity was also due to differences in host defense. As the multi-step growth of C-PA in Vero cells was better than that of WSN, and because its single-step growth, which is highly dependent on the polymerase activity, in both MDCK and Vero cells was better than that of WSN (Figure 3), we first examined the activity of the interferon β promoter and found that it was strongly activated (Figure 5) in CPA-infected cells.

Type I interferon is induced in virus-infected cells by a signal transduction pathway beginning with retinoic-acid-inducible gene-I (RIG-I), which recognizes 5′-triphosphate-containing influenza RNA [57-62]. In C-PA-infected cells, a large amount of vRNA was expressed from early stages of infection, which might more strongly trigger interferon induction (Figures 5 and Additional file 1: Figure S3) and thereby induce an antiviral state in uninfected cells that protected them from influenza infection (Additional file 1: Figure S2A) [63-65]. The better multi-step growth of C-PA relative to WSN in Vero cells, which are defective in type-I interferon production [66] (Figure 2C), and the better single-step growth of C-PA than of WSN (Figure 3), are consistent with the high *in vitro* polymerase activity and strong interferon induction in C-PA infected cells.

Apoptosis (programmed cell death) is another mechanism by which cells restrict viral infection including influenza [67]. However, virus-induced apoptosis causes tissue damage, which is one of the mechanisms of influenza pathogenicity [68]. Apoptosis also aids in the release of influenza viruses [69,70]. WSN induced apoptosis in MDCK cells late in infection [71]. However, no apoptosis was induced by expression of any single protein of either WSN or H5N1 Cambodia. Apoptosis was strongly induced in C-PA-infected cells beginning early in infection when no apoptosis was induced in WSN-infected cells (Figure 6). The activation of caspase 9 indicated that this apoptosis was mediated through the mitochondrial pathway [72]. The increased expression of vRNA (Additional file 1: Figure S3) in C-PA infected cells due to the high replication activity promoted by H5N1 PA (Figure 1) induced both interferon and apoptosis, resulting in attenuation of C-PA proliferation (Figure 2). Neither caspase 3 nor 8 was induced by expression of WSN PA or Cambodia PA alone (data not shown). The strong induction of interferon discussed above also stimulates the induction of apoptosis *via* RNA-dependent protein kinase (PKR) [73]. Such rapid induction of apoptosis was also observed in duck cells infected with HPAIV [68]. However, in case of C-PA, early induction of apoptosis attenuated the proliferation of the virus and thus decreased its pathogenicity.

PB1-F2 is the only influenza virus protein that induces apoptosis in infected monocytes and potentiates apoptosis during infection [74-76]. The WSN PB1-F2 proteins of WSN and C-PA are identical. Kinetic analysis of viral RNAs ( 1: Figure S 3) indicates that PB1-F2 is unlikely to contribute to the strong induction of apoptosis in C-PA infected cells.

C-PA infection strongly induced both interferon production and apoptosis early in infection, which attenuated virus proliferation and pathogenicity despite high RdRp activity. This may be another reason, in addition to poor adaptation, for the difficulty of obtaining reassortant viruses carrying H5N1 HPAIV PA [31,32].

## Methods

### Cell culture

293 T and Vero cells were maintained in Dulbecco’s modified Eagle minimal essential medium (DMEM) containing 10% fetal bovine serum (FBS). CEFs were prepared from 11-day-old embryonated chicken eggs by trypsin digestion and stored in liquid nitrogen. CEF and MDCK cells were maintained in DMEM containing 10% or 5% FBS, respectively.

### Replicon assay

A standard replicon assay was performed as previously described [41,42,77]. Luciferase activity was measured using the Dual-Glo luciferase assay kit and a GloMax 96 Microplate Luminometer (Promega, Fitchburg, USA) after transfection of 0.1 μg each of pCPB2, pCPB1, pCPA, pCNP, and ppolINSluc and 0.01 μg of pRL_SV40_ (Promega) using Lipofectamine 2000 (Invitrogen, Carlsbad, USA). The PB2, PB1, PA, and NP genes of influenza A/Cambodia/P096/2005 (H5N1) [40] and A/WSN/33 (H1N1) [56] were PCR-amplified using specific oligonucleotides and cloned into pcDNA3.1(+) (Invitrogen), resulting in pcCamPB2wt, pcCamPB1, pcCamPA, pcCamNP, pcWSNPB2, pcWSNPB1, pcWSNPA, and pcWSNNP, respectively. Only data in which the variation of the Renilla luciferase activity was less than 3-fold variations were used. The oligonucleotide sequence information will be made available upon request.

### Expression, purification, and *in vitro* transcription of the influenza virus RdRp

Expression, purification, and *in vitro* transcription of the influenza virus RdRp were performed as previously described [42,77,78]. Briefly, 100 nM influenza RdRp was incubated in 50 mM Tris–HCl (pH 8.0), 8 mM MgCl_2_, 150 mM NaCl, 2 mM DTT, 0.5 mM ATP, 0.5 mM CTP, 0.5 mM GTP, 0.05 mM [α-^32^P] UTP, 0.1 mM ApG or 0.01 mg/mL globin mRNA, 2000 U/mL RNase inhibitor, and 200 nM v84 and c84 model template RNA at 25°C for 90 min. The product was analyzed by PAGE on 6% gels containing 8 M urea and the images analyzed with a Typhoon Trio plus (GE Healthcare, Bucks, UK).

### Model RNA templates

v84 and c84 model RNA templates were prepared as previously reported [11].

### Western blotting

Proteins were blotted onto nitrocellulose membranes (Millipore, Billerica, USA) by semi-dry electroblotting (Bio-Rad, Hercules, USA) after SDS-PAGE on 10% gels. The blotted membranes were blocked with 10% skim milk in 20 mM Tris–HCl (pH 7.5), 150 mM NaCl, and 0.02% Tween 20 (TBST), and western blotting was performed using rabbit anti-PB1, -PB2, -PA [11], and -PR8 (1:1,000 each) and anti-actin (1:100) antibodies as the primary antibodies. The membranes were then incubated with alkaline phosphatase-conjugated anti-rabbit IgG (1:7,500) or anti-mouse IgG (1:7,500), and the positions of the bound antibodies were visualized with nitroblue tetrazolium (NBT) and 5-bromo-4-chloro-3-indolyl phosphate (BCIP).

### Reconstitution of influenza virus and plaque-formation assay

pHH21 and the influenza virus 12-plasmid reconstitution system of WSN were kindly provided by Dr. Hobom and Dr. Kawaoka [56]. The H5N1 A/Cambodia/P0322095/2005 [40] PA sequence was inserted into ppolI-WSN-PA, resulting in ppolI-Cam-PA. The influenza virus WSN strain (WSN) and WSN carrying H5N1 Cambodia PA (C-PA) were reconstituted by co-transfection of 293 T cells with ppolI-WSN-PB2, ppolI-WSN-PB1, ppolI-WSN-PA or ppolI-Cam-PA, ppolI-WSN-HA, ppolI-WSN-NP, ppolI-WSN-NA, ppolI-WSN-M, and ppolI-WSN-NS with pCPB2, pCPB1, pCPA, and pCNP [10,56] using Lipofectamine 2000. The reconstituted viruses were recovered from the culture media 72 hr post-transfection. The viruses were plaque-purified, amplified, and titered on MDCK cells by a plaque-formation assay and stored at −80°C. The genome sequences of all viruses were determined by RT-PCR. The primer sequences used will be made available upon request.

### Virus growth assay

MDCK, CEF, and Vero cells in 3.5-cm dishes (21 dishes of MDCK cells and CEFs, 27 dishes of Vero cells per a virus) were infected with WSN and C-PA at an MOI of 0.01 at 37°C for 1 hr (multi-step growth assay). The cells were washed 3× with phosphate-buffered saline (PBS) and incubated with 2 mL of DMEM containing 2% FBS (without trypsin) at 37°C, as WSN replicates without trypsin [79]. All of the supernatants of 3 dishes of each cell were taken 6, 12, 24, 36, 48, and 60 hr (and also at 72 and 90 hr for Vero cells) after infection and stored at −80°C. Virus growth was also tested in MDCK and Vero cells after infection at an MOI of 5 (single-step growth assay). Supernatants were harvested 2, 4, 6, 8, 10, and 12 hr after infection. The viruses in the supernatants were titered on MDCK cells by a plaque-formation assay.

### Mouse infection

Groups of 4 6-week-old female BALB/c mice (Sino-British Laboratory Animal, Shanghai, China) were anesthetized with ether and inoculated with 10^5^, 10^4^, or 10^3^ PFU of WSN or 10^6^, 10^5^, 10^4^, or 10^3^ PFU of C-PA in a volume of 50 μL by nasal dropping. Four mice were inoculated with 50 μL of PBS as a mock-infection control. The survival rates were monitored daily and the 50% lethal doses (LD_50_s) calculated [41].

### Histopathological analysis

Six-week-old female BALB/c mice were anesthetized with ether and inoculated with 10^5^ PFU of WSN or C-PA in 50 μL by nasal dropping. Mice were inoculated with 50 μL of PBS as a mock-infection control. A non-treated mouse was used as a non-treated control. The mock-infected and infected mice were sacrificed by cervical dislocation under anesthesia on days 1, 2, 3, and 4 after infection, and their lungs were removed and fixed with 3.5% formalin/PBS at 25°C for 2 days. The lungs were embedded in paraffin blocks, sectioned at 4-μm thickness, and stained with hematoxylin and eosin (HE).

### TUNEL assay

Cells were placed on cover slips in 24-well plates and infected with WSN or C-PA at an MOI of 0.01. Eight, 9, 10, 11, 12, 13, 14, 15, 16, and 24 hr after infection, cells were fixed with 1% formalin/PBS. The TUNEL assay was performed using the DeadEnd^TM^ Fluorometric TUNEL system according to the company’s instructions. The samples were observed using a fluorescence microscope (Leica DM IRB, Leica, Wetzlar, Germany), and the numbers of TUNEL positive cells in 3 random fields were counted.

### Caspase activity

MDCK cells were plated in 10-cm-diameter plates and infected with WSN or C-PA at an MOI of 0.01. 10 hr after infection, the cells were harvested and the activities of caspases 3, 8, and 9 measured using the Caspase-3/CPP32 Fluorometric Assay kit, the Caspase 8/FLICE Fluorometric Assay kit, and the Caspase 9 Fluorometric Assay kit (Biovision, Inc., Milpitas, USA) according to the manufacturer’s instructions.

### Interferon induction

Interferon induction was analyzed by measuring the luciferase activity of cells transfected with p-125Luc, which was kindly provided by Dr. Fujita [80]. 293 T cells were transfected with p-125Luc (1 μg) and pRL_SV40_ (100 ng). Four hours post-transfection, the cells were infected with WSN or C-PA at an MOI of 0.01. The interferon promoter activity was measured as the luciferase activity using the Dual-Glo luciferase assay kit and a GloMax 96 Microplate Luminometer (Promega) and normalized as the firefly luciferase/Renilla luciferase activity ratio.

### Chemicals and radioisotopes

Non-radiolabeled nucleotides were purchased from GE Healthcare, [α-^32^P]UTP from New England Nuclear (PerkinElmer Life Sciences, Waltham, USA), and T7 RNA polymerase, T4 nucleotide kinase, oligonucleotides, human placental RNase inhibitor, and restriction enzymes from Takara (Dalian, China). The RPAIII Ribonuclease Protection Assay Kit was purchased from Ambion (Austin, USA). Anti-actin antibodies were purchased from Sigma Aldrich (St. Louis, USA). The Dual-Glo luciferase assay kit, DeadEnd^TM^ Fluorometric TUNEL system, alkaline phosphatase-conjugated anti-rabbit and anti-mouse IgG’s, NBT, and BCIP were purchased from Promega. DMEM, FBS, Lipofectamine 2000, and Trizol reagent were purchased from Invitrogen.

### Statistical analysis

The statistical significance levels of the data were evaluated by Student’s *t*-test, with p < 0.05 indicating statistical significance.

## Competing Interests

The authors declare that they have no competing interests.

## Authors’ contributions

QW, SZ, HJ, JW, LW, YM, and KX carried out the experiments. SS, FY, and MK analyzed caspase activity and mouse lung tissues. PB and VD isolated and cloned HPAIV H5N1 Cambodia. QW, SZ, SB, and TT conceived of the study. QW and SZ analyzed the data and drafted the manuscript, and SB and TT reviewed the manuscript. All authors read and approved the final manuscript.

## Supplementary Material

Additional file 1 **Results, Methods. Figure S1.** Expression of RdRp subunits and NP in the transfected cells**. Figure S2.** Time course of NP protein expression by C-PA and WSN. **Figure S3.** Time courses of NP vRNA, cRNA, and mRNA expression by C-PA and WSN. Click here for file
