# Supplementary material for: PA from an H5N1 highly pathogenic avian influenza virus activates viral transcription and replication and induces apoptosis and interferon expression at an early stage of infection
Source: Virol J. 2012 Jun 8;9:106. doi: 10.1186/1743-422X-9-106 (PMC3507744; doi:10.1186/1743-422X-9-106)
Supplement: Additional file 1 — Results, Methods. Figure S1. Expression of RdRp subunits and NP in the transfected cells. Figure S2. Time course of NP protein expression by C-PA and WSN. Figure S3. Time courses of NP vRNA, cRNA, and mRNA expression by C-PA and WSN. [file 1743-422X-9-106-S1.pdf]

Additional file

PA from an H5N1 highly pathogenic avian influenza virus activates viral transcription and replication, and induces apoptosis and interferon expression at early stage of infection

Qiang Wang, Shijian Zhang, Hongbing Jiang, Jinlan Wang, Leiyun Weng, Yingying Mao, Satoshi Sekiguchi, Fumihiko Yasui, Michinori Kohara, Philippe Buchy, Vincent Deubel, Ke Xu, Bing Sun, Tetsuya Toyoda

Results

NP expression

We analyzed the protein expression in the infected MDCK cells by kinetic quantitative western blotting against NP (Fig. S2). NP expression at 8 hr post-infection (pi) was higher in C-PA-infected cells than in WSN-infected cells ($p < 0.05$), while the amounts were similar 16 hr pi. NP expression 24 hr pi was almost twice as high in WSN-infected cells as in C-PA-infected cells ($p < 0.01$). The actin signal was no longer detectable in WSN-infected cells 36 hr pi because of cell damage, whereas it remained

detectable in C-PA-infected cells. The retained actin signal in C-PA-infected cells indicates that many cells survived the C-PA infection (this experiment was performed at a multiplicity of infection (MOI) of 0.01).

The amount of NP 4 hr pi at an MOI of 1 was measured because no clear NP signal was detected at an MOI of 0.01. At an MOI of 1, there was no clear difference between C-PA and WSN in the amount of NP expressed 4 hr pi (data not shown).

Quantitation of NP mRNA, cRNA, and vRNA

We compared the amounts of NP vRNA, cRNA, and mRNA in WSN- and C-PA-infected cells using an RNase protection assay (Fig. S3). The vRNA signal accumulated in C-PA-infected cells in proportion to the time post-infection and was significantly larger than that in WSN-infected cells. The amounts of cRNA 4, 8, and 16 hr pi were similar between C-PA- and WSN-infected cells. The mRNA signals 4, 8, and 16 hr pi were similar in C-PA-infected cells, while the mRNA signals 4 and 8 hr pi were significantly larger in WSN-infected cells than in C-PA-infected cells. The amount of mRNA in WSN-infected cells decreased in proportion to the time post-infection.

The NP mRNA levels 1 and 2 hr pi were analyzed in cells infected at an MOI of 1 because no signal was detected after infection at an MOI of 0.01. There was no significant difference between C-PA- and WSN-infected cells (Inset of Fig. S3).

Methods

Antibodies against influenza virus PR8

Antibodies against the PR8 strain were produced by immunization of a rabbit with purified PR8 virions.

Quantitation of NP protein by western blotting

The expression levels of NP protein in WSN- and C-PA-infected MDCK cells were compared by western blotting. Cells were infected with viruses at an MOI of 0.01 and harvested 8, 16, 24, and 36 hr pi. The image of the blot was scanned with an image scanner (Epson ES-8500, Epson, Tokyo, Japan), and the signals were quantified with Image J 1.44P (http://imagej.nih.gov/ij).

Quantitation of NP RNA by RNase protection assay

The expression levels of NP vRNA, cRNA, and mRNA in WSN- and C-PA-infected MDCK cells were compared using an RPAIII Ribonuclease Protection Assay Kit. Total RNA from cells infected at an MOI of 0.01 was extracted 2, 4, 8, and 16 hr pi using Trizol, and 0.1 mg of total RNA was analyzed. For detection of vRNA (180-nt fragment), the positive-sense sequence of the NP gene (nt 1,386–1,565), and for detection of mRNA (194-nt fragment) and cRNA (216 nt fragment), the negative-sense sequences of NP gene (nt 1,350–1,565) were labeled with [α-^{32}P]UTP using T7 RNA

polymerase.

Supplementary figure legends.

Figure S1. Expression of RdRp subunits and NP in the transfected cells.

The expression levels of the RdRp subunits (PB2, PB1, and PA) and NP in transfected 293T cells were confirmed by western blotting with anti-PB2, -PB1, -PA, and -PR8 antibodies. The same volume of cell lysate (10 μL) was analyzed in each case.

Figure S2. Time course of NP protein expression by C-PA and WSN

The expression of NP protein in cells infected with C-PA or WSN was measured by western blotting. MDCK cells were infected with C-PA or WSN at an MOI of 0.01. Cells were harvested 8, 16, 24, and 36 hr pi and processed for western blotting with anti-PR8 and anti-actin antibodies (A). The position of the pre-stained protein standard marker (M) is indicated on the left. The average ratio of NP to actin and its standard deviation (error bar) were calculated from 3 independent experiments (B). Statistical significance was evaluated with Student's *t*-test. *$p < 0.05$, **$p < 0.01$

Figure S3. Time courses of NP vRNA, cRNA, and mRNA expression by C-PA and

WSN

The expression levels of NP vRNA, cRNA, and mRNA in MDCK cells infected with C-PA or WSN at an MOI of 0.01 were measured by an RNase protection assay. Cells infected with C-PA or WSN were harvested 4, 8, and 16 hr pi, and the total RNA was extracted. RNA (0.1 mg) was processed for RNase protection. Inset: The amounts of NP mRNA expressed 1 and 2 hr pi at an MOI of 1. The mean signals of NP vRNA, cRNA, and mRNA and their standard deviations (error bars) were calculated from 3 independent experiments subjected to PAGE on the same gel. Statistical significance was evaluated with Student's *t*-test. *$p < 0.05$, **$p < 0.01$

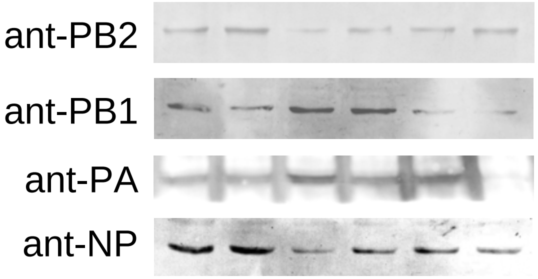

PB2	P	P	W	W	C	P
PB1	P	P	W	W	C	P
PA	P	C	W	C	C	-
NP	P	P	W	W	C	P

Fig. S1. Wang

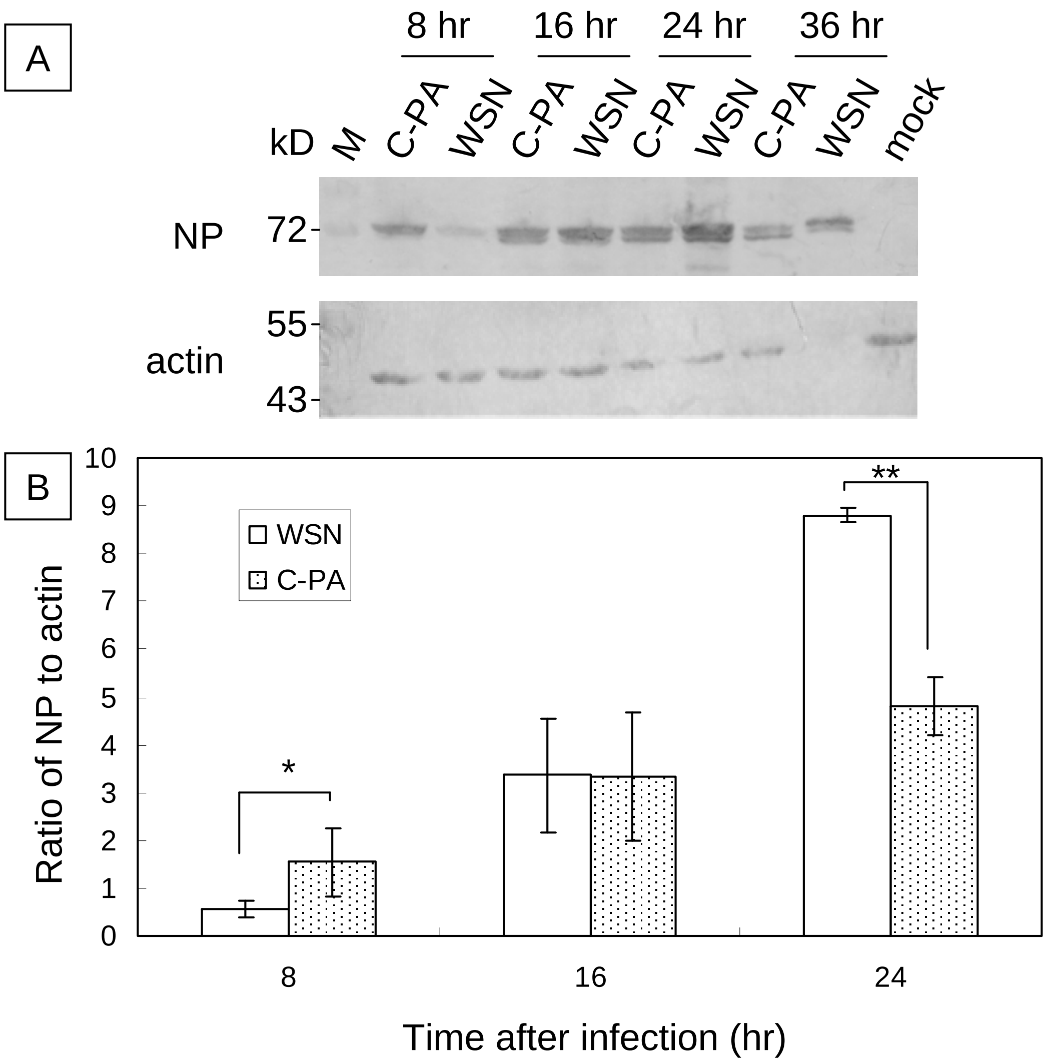
A
8 hr
16 hr
24 hr
36 hr
kD
M
C-PA
WSN
C-PA
WSN
C-PA
WSN
C-PA
WSN
mock
NP
72-
55-
actin
43-
B
WSN
C-PA
*
**
Ratio of NP to actin
10
9
8
7
6
5
4
3
2
1
0
8
16
24
Time after infection (hr)

Fig. S2. Wang

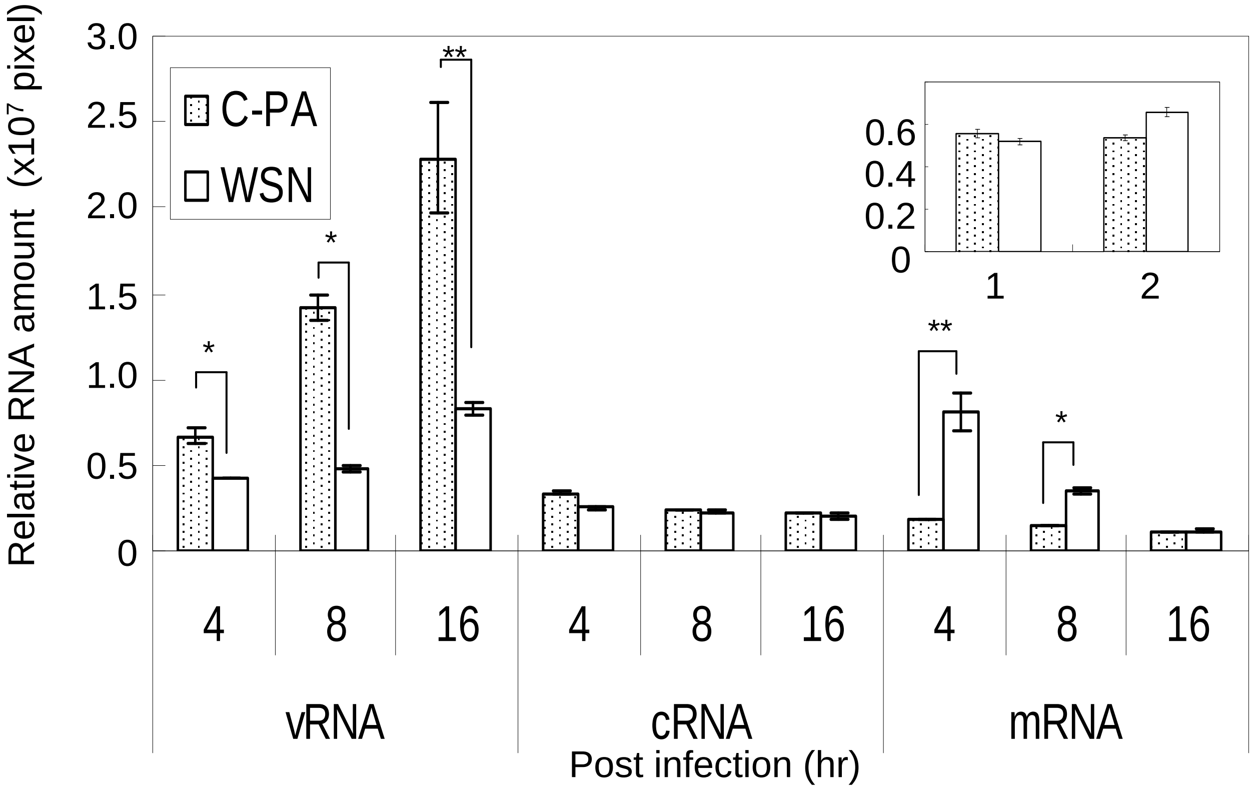

Relative RNA amount (x10^7 pixel)
3.0
2.5
2.0
1.5
1.0
0.5
0
C-PA
WSN
**
*
*
**
*
0.6
0.4
0.2
0
1
2
4
8
16
4
8
16
4
8
16
vRNA
cRNA
mRNA
Post infection (hr)

Fig. S3. Wang
